# Supplementary material for: Exploring the Perceptions of Undergraduate Pharmacy Students’ Communication Skills to Facilitate Better Professional Decision-Making in the UK
Source: Pharmacy (Basel). 2025 Aug 28;13(5):117. doi: 10.3390/pharmacy13050117 (PMC12452427; doi:10.3390/pharmacy13050117)
Supplement: Supplementary file 1 [file pharmacy-13-00117-s001.zip › pharmacy-3720673-supplementary.pdf]

medway school of pharmacy

Exploring the perceptions of undergraduate pharmacy students’ communication skills

Participant Information Leaflet

Pharmacy professionals have an important role in the delivery of patient-centred care, with effective communication and the consultation process forming the foundation of interactions with patients and healthcare professionals.

Please take some time to read this participant information leaflet which provides you information about this study.

What is the purpose of the study?

With the evolving roles of pharmacy professionals and developments in virtual consultations, pharmacy students need to have high-quality communication skills. The pharmacy regulatory body in the UK, the General Pharmaceutical Council (GPhC), sets out standards for pharmacy professionals in which effective communication is a priority to deliver safe and effective care. Pharmacy schools in the UK offer an undergraduate Master of Pharmacy (MPharm) degree which is currently accredited by the GPhC and communication skills are integral to the curriculum. Therefore, pharmacy schools play a key role in incorporating this crucial skill when developing pharmacy students and future pharmacists. Changes in UK pharmacy education have focused on the increased clinical aspect of pharmacy practice, including communication skills, but it is not known how this has affected pharmacy students. We would like to discover the communication skills training provided in UK pharmacy schools. In addition, we would like to explore the perceptions of undergraduate pharmacy students’ communication skills.

Do I have to take part?

No. It is entirely your decision whether you decide to take part in this study or not. We would like you to take part in this study if you are an undergraduate pharmacy student, over 18 years of age, undertaking an undergraduate Master of Pharmacy (MPharm) degree in a University in the UK. If you decide to take part, you can change your mind at any time. However, once you a have submitted the online survey, we will not be able to withdraw your responses. This is because the survey is anonymous, therefore, we will not be able identify which response is yours.

If I decide to take part, what do I need to do?

If you decide to take part in this study, you will be asked to complete the online survey for this study. It will take up to 10 minutes to complete and your responses will be anonymised.

Are there any benefits if I take part?

All participants will be able to enter into an optional prize draw to receive one of five £20 Amazon vouchers. The draw will be made at the end of the study in April 2023.

Are there any risks if I take part?

There are no identifiable risks if you take part in the study. If you choose to enter into the optional prize draw, please be assured that your contact details will not be linked to your survey responses and your survey responses will remain anonymous. Contact details will be stored securely by the principal investigators (Dr Sukvinder Kaur Bhamra and Mr Aadesh Dave) on password-protected software and they will be deleted immediately after the prize draw.

Will anyone know that I've taken part?

No one will be told about your participation in this study. No personal identifiable data will be collected. All results will be anonymous.

What will happen to the results?

The data from the survey will be analysed and published as part of the sustained research projects for the undergraduate pharmacy students' thesis, and potentially in academic journals and conferences. Anonymised data from the survey will be kept for twelve months after the study is completed, after which the data will be deleted.

If you wish to be provided with information about the study findings please provide your contact details to Dr Sukvinder Kaur Bhamra at s.k.bhamra@kent.ac.uk or Mr Aadesh Dave at a.dave@kent.ac.uk and you will be sent a copy of the findings.

Who is organising and funding the study?

This study is funded by the Medway School of Pharmacy at Universities of Greenwich and Kent. It is being carried out by a research team led by Dr Sukvinder Kaur Bhamra and Mr Aadesh Dave who are both registered pharmacists and lecturers.

Who should I contact if I want to know more about the study?

If you want to know more about the study, please contact the principal investigators, Dr Sukvinder Kaur Bhamra at s.k.bhamra@kent.ac.uk or Mr Aadesh Dave at a.dave@kent.ac.uk.

Who should I contact if I have any concerns about the study or the way it has been conducted?

If you have concerns about how this research study has been conducted, please contact the Chair of the Medway School of Pharmacy Research Ethics Committee, Dr Barbra Katusiime at b.katusiime@kent.ac.uk.

If you would like to know more about the Universities guidance on the use of personal data, it can be found here: https://research.kent.ac.uk/researchservices/wp-content/uploads/sites/51/2018/05/GDPR-Privacy-Notice-Research.pdf.

Who has reviewed this study?

The Medway School of Pharmacy Research Ethics Committee have reviewed and approved this study; they have given permission for this study to take place. This does not mean that you have to take part in this study, it is completely your decision.

Thank you for taking time to consider taking part in this study.

* 1. By completing and submitting your responses to this survey, you are giving your consent to be part of this study and for your data to be used as described in the participant information leaflet.

Please confirm your consent to take part in this study by completing this online survey:

- ◯ Yes, I consent to take part
- ◯ No, I do **not** consent to take part

medway school of pharmacy

Exploring the perceptions of undergraduate pharmacy students’ communication skills

* 2. Are you currently an undergraduate pharmacy student, over 18 years of age, undertaking an undergraduate Master of Pharmacy (MPharm) degree in a University in the UK (i.e. England, Scotland, Wales or Northern Ireland)?

◯ Yes, I am

◯ No, I am **not**

medway school of pharmacy

Exploring the perceptions of undergraduate pharmacy students’ communication skills

Section 1 - About You

3. What is your current school of pharmacy?

- ◯ Aston University (Birmingham)
- ◯ University of Bath
- ◯ University of Birmingham
- ◯ University of Bradford
- ◯ University of Brighton
- ◯ Cardiff University
- ◯ University of Central Lancashire (Preston)
- ◯ University College London
- ◯ De Montfort University (Leicester)
- ◯ University of East Anglia (Norwich)
- ◯ University of Hertfordshire (Hatfield)
- ◯ University of Huddersfield
- ◯ Keele University
- ◯ King's College, University of London
- ◯ Kingston University London
- ◯ University of Lincoln
- ◯ Liverpool John Moores University
- ◯ Medway School of Pharmacy, Universities of Greenwich and Kent
- ◯ University of Manchester
- ◯ Newcastle University
- ◯ University of Nottingham
- ◯ University of Portsmouth
- ◯ Queen’s University Belfast
- ◯ University of Reading
- ◯ Robert Gordon University (Aberdeen)
- ◯ University of Strathclyde (Glasgow)
- ◯ University of Sunderland
- ◯ Swansea University
- ◯ Ulster University (Coleraine)
- ◯ University of Wolverhampton
- ◯ Other, please specify: ______________________

4. What undergraduate MPharm degree programme are you currently registered on?

- 4 Year MPharm
- 5 Year MPharm
- MPharm Extended
- Other, please specify:

5. What is your current year of study?

- Year 0 (Undergraduate Foundation Year)
- Year 1
- Year 2
- Year 3
- Year 4
- Year 5 (Integrated Pre-registration/Foundation Training)
- Other, please specify:

medway school of pharmacy

Exploring the perceptions of undergraduate pharmacy students' communication skills

6. What is your gender?

- Female
- Male
- Non-Binary
- Prefer not to say
- Other, please specify:

7. What is your age?

- ◯ 18-20
- ◯ 21-30
- ◯ 31-40
- ◯ 41-50
- ◯ 51+

8. What is your ethnicity?

- ◯ White: British
- ◯ White: Irish
- ◯ White: Other
- ◯ Black: African/Caribbean
- ◯ Black: Other
- ◯ Arab
- ◯ Asian: Indian
- ◯ Asian: Pakistani
- ◯ Asian: Bangladeshi
- ◯ Asian: Chinese
- ◯ Asian: Other
- ◯ Mixed Race
- ◯ Other, please specify:

9. Is English your first language?

- ◯ Yes
- ◯ No

medway school of pharmacy

Exploring the perceptions of undergraduate pharmacy students' communication skills

Section 2 - Communication Skills

10. For each of the following statements about your communication skills, select the most suitable option:

	Strongly Disagree	Disagree	Unsure	Agree	Strongly Agree
I have received **theoretical** training in communication skills from my undergraduate MPharm degree	○	○	○	○	○
I have received **practical** training in communication skills from my undergraduate MPharm degree	○	○	○	○	○
I have received **feedback** on my communication skills from my undergraduate MPharm degree	○	○	○	○	○

11. For each of the following statements about your confidence in specific scenarios, select the most suitable option:

	Very Unconfident	Unconfident	Unsure	Confident	Very Confident
I can introduce myself to my peers	○	○	○	○	○
I can introduce myself to a patient	○	○	○	○	○
I can counsel a patient on their medication	○	○	○	○	○
I can discuss, with any healthcare professional, my concerns if the dose of a medication is too high for a patient and provide suitable recommendations	○	○	○	○	○

12. How would you rate your communication skills? (1 = poor and 5 = excellent)

1	2	3	4	5
○	○	○	○	○

13. Which of the following statements best describes your communication skills?

- ◯ My communication skills are linked to my personality
- ◯ My communication skills are a result of the training from my undergraduate MPharm degree
- ◯ My communication skills are due to my extracurricular work experiences/activities
- ◯ None of the above statements best describe my communication skills

14. Do you believe your communication skills can be improved?

- ◯ Yes*
- ◯ No
- ◯ Unsure

*If yes, please specify in which areas you believe your communication skills can be improved and what additional training you may require:

medway school of pharmacy

Exploring the perceptions of undergraduate pharmacy students' communication skills

Section 3 - Communication Skills Training

15. In what year(s) are communication skills taught in your undergraduate MPharm degree? (select all that apply)

- ☐ Year 0 (Undergraduate Foundation Year)
- ☐ Year 1
- ☐ Year 2
- ☐ Year 3
- ☐ Year 4
- ☐ Year 5 (Integrated Pre-registration/Foundation Training)
- ☐ Other, please specify:

16. What communication skills teaching methods(s) are included in your undergraduate MPharm degree? (select all that apply)

- ☐ Practice with real patients
- ☐ Role play with teachers/academics
- ☐ Role play with simulated patients
- ☐ Role play with peers
- ☐ Observing other healthcare professionals
- ☐ Observing teachers/academics
- ☐ Video recording of consultations
- ☐ Feedback from teachers/academics
- ☐ Lectures
- ☐ Online learning materials
- ☐ Books, literature or course learning materials
- ☐ Reflective writing
- ☐ Problem-Based Learning (PBL)
- ☐ Team-Based Learning (TBL)
- ☐ Small group teaching (e.g. Workshops)
- ☐ Placements
- ☐ Other, please specify:

17. Following on from Question 16, rank the top **five** teaching methods(s) you believe are the most effective to learn communication skills (1 = most effective and 5 = least effective):

	1	2	3	4	5
Practice with real patients	○	○	○	○	○
Role play with teachers/academics	○	○	○	○	○
Role play with simulated patients	○	○	○	○	○
Role play with peers	○	○	○	○	○
Observing other healthcare professionals	○	○	○	○	○
Observing teachers/academics	○	○	○	○	○
Video recording of consultations	○	○	○	○	○
Feedback from teachers/academics	○	○	○	○	○
Lectures	○	○	○	○	○
Online learning materials	○	○	○	○	○
Books, literature or course learning materials	○	○	○	○	○
Reflective writing	○	○	○	○	○
Problem-Based Learning (PBL)	○	○	○	○	○
Team-Based Learning (TBL)	○	○	○	○	○
Small group teaching (e.g. Workshops)	○	○	○	○	○
Placements	○	○	○	○	○

Other, please specify and provide a ranking:

[ ]

18. Have you had the opportunity to undertake interprofessional education (learning together with students of other healthcare professions) in your undergraduate MPharm degree?

- ◯ Yes*
- ◯ No
- ◯ Unsure

*If yes, please specify which healthcare professionals you have undertaken interprofessional education with in your undergraduate MPharm degree:

medway school of pharmacy

Exploring the perceptions of undergraduate pharmacy students' communication skills

19. For the following statement about interprofessional education, please select the most suitable option:

	Strongly Disagree	Disagree	Unsure	Agree	Strongly Agree
I believe interprofessional education has improved my communication skills	◯	◯	◯	◯	◯

medway school of pharmacy

Exploring the perceptions of undergraduate pharmacy students' communication skills

Section 4 - Professional Decision-Making Skills

Professional Decision-Making: The process in which a healthcare professional chooses the best course of action about care after carefully analysing and examining the available options and the given circumstances.

20. For each of the following statements about professional decision-making skills, please select the most suitable option:

	Strongly Disagree	Disagree	Unsure	Agree	Strongly Agree
I believe I can appropriately make professional decisions	○	○	○	○	○
I believe my communication skills have facilitated my professional decision-making skills	○	○	○	○	○
I believe my communication skills **training** has facilitated my professional decision-making skills	○	○	○	○	○

medway school of pharmacy

Exploring the perceptions of undergraduate pharmacy students’ communication skills

Section 5 - Additional Comments

21. Please provide any additional comments or thoughts about your communication skills or this survey:

medway school of pharmacy

Exploring the perceptions of undergraduate pharmacy students’ communication skills

Contact Details for Prize Draw (Optional)

If you would like to be entered into a prize draw for a chance to win one of five £20

Amazon vouchers, please confirm your consent and leave your contact details.

Your personal details will not be associated with your responses from this survey. Please be assured your responses will remain anonymous. Your personal details will only be used to contact you if you win an Amazon voucher.

22. Please confirm your consent to be contacted for the following purposes:

- ◯ I consent that I would like to be entered into the prize draw and be contacted if I win
- ◯ I would **not** like to be entered into the prize draw and be contacted

23. Contact details:

Full name: ____________________

Email address: ____________________
